# Mycorrhizal Fungal Partners Remain Constant during a Root Lifecycle of *Pleione bulbocodioides* (Orchidaceae)

**DOI:** 10.3390/jof7110994

**Published:** 2021-11-22

**Authors:** Jiao Qin, Jing-Qiu Feng, Wei Zhang, Shi-Bao Zhang

**Affiliations:** 1 Key Laboratory of Economic Plants and Biotechnology, Kunming Institute of Botany, Chinese Academy of Sciences, Kunming 650201, China; qinjiao@mail.kib.ac.cn (J.Q.); fengjingqiu@mail.kib.ac.cn (J.-Q.F.); zhangwei@mail.kib.ac.cn (W.Z.); 2 Yunnan Key Laboratory for Fungal Diversity and Green Development, Kunming 650201, China; 3 Yunnan Key Laboratory for Wild Plant Resources, Kunming 650201, China; 4 University of Chinese Academy of Sciences, Beijing 100049, China

**Keywords:** endophytic fungi, epiphytic orchid, Mi-Seq, mycorrhizal fungi, pseudobulb, root age, Shancigu

## Abstract

Mycorrhizal mutualisms are vital for orchids through germination to adulthood. Fungal species diversity and community composition vary across seasons and plant development stages and affect plant survival, adaptation, and community maintenance. Knowledge of the temporal turnover of mycorrhizal fungi (OMF) remains poorly understood in the eco-physiologically diverse orchids (especially in epiphytic orchids), although it is important to understand the function and adaptation of mycorrhizae. Some species of *Pleione* are epiphytic plants with annual roots and may recruit different fungal partners during their root lifecycle. Based on continuous samplings of *Pleione bulbocodioides* during a whole root lifecycle, we characterized the fungal temporal dynamics using Illumina sequencing of the ITS2 region. Our data showed that the plants of *P. bulbocodioides* were quickly colonized by OMF at root emergence and had a constant OMF composition throughout one root lifecycle, although the OMF richness declined with root aging after a peak occurrence during root elongation. In contrast, the richness of root-inhabiting fungal endophytes kept increasing with root aging and more drastic turnovers were found in their species compositions. Our findings of OMF temporal turnover contribute to further understanding of mycorrhizal associations and adaptation of Orchidaceae and will benefit orchid resource conservation and utilization.

## 1. Introduction

Fungal associates provide plants with a range of benefits and play crucial roles in plant survival and diversifications [1]. Widespread intraspecific heterogeneity in fungal symbiotic interactions (along a continuum from specialism to generalism) has been frequently observed due to spatial variations in ecological conditions [2]. Common temporal changes within symbioses, ranging from lifelong fidelity to total replacement of fungal partners, also exist because plant physiological demands and ecological factors can also change over time. Temporal turnovers of mycorrhizal fungi associated with different plants have drawn wide attention because of the importance in understanding functions and adaptations of mycorrhizae [3,4]. To date, changes in fungal richness and compositions have been detected in most mycorrhizal interaction types, including ericoid, arbuscular, ecto-, and orchid mycorrhizal relationships [5,6,7,8].

Fungal interactions are critical for orchids because their dust-like seeds that contain only minimal nutrient reserves, cannot develop to the seedling stage without the establishment of an association with orchid mycorrhizal fungi (OMF) [9]. Seed germination and protocorm development of orchids require organic carbon provided by fungal partners [10,11]. Adult orchids remain mycorrhizal and are thought to rely on fungal associations for mineral uptake and often for a supplement of carbon [12,13]. The intrinsic factors, viz., different physiological needs between orchid ontogenetic stages (germination and adulthood or vegetative and reproductive stages), may cause changes in the cost–benefit balance of mutualisms and thereby provoke shifts in their OMF recruiting [14,15]. In recent years, turnover of OMF partners has been repeatedly reported from juveniles to adults of species within Orchidaceae [16,17,18,19].

Extrinsic or environmental drivers, e.g., seasonal changes in temperature or water/resource availability or the degree of environmental stress for both plants and fungi, may also cause shifts in fungal symbiosis [20,21,22,23]. Changes in OMF richness and compositions at annual or seasonal scales have also been reported in a few species [8,24,25]. The temporal turnover of OMF in different plant taxa can be categorized into 11 different scenarios that vary in a broad range from fidelity to replacement across time points [3]. The scenarios of OMF temporal turnover and possible driving factors are not fully understood in Orchidaceae since the taxa and eco-physiological performances are extremely abundant and diverse in this family.

Many species in the Orchidaceae family are terrestrial and have subterranean roots or tubers, while over 70% of the members of this family are epiphytic (including lithophytic) and their roots are usually exposed on the surface of rocks or tree stems [26]. Similar with the terrestrial orchids, the epiphytic species also have persistently present OMF colonization in their roots [27]. Different plant taxa and their mycorrhizal fungi may have various physiological demands (e.g., in water and nutrition utilization) [28]. The seasonal environmental changes can affect fungal richness, composition, and activities in the soil pool [21,22,23]. The fluctuation of soil fungi may directly affect or act synergistically with intrinsic factors on OMF selection of the terrestrial species within Orchidaceae. The water and nutrient availabilities in epiphytic habitats (e.g., on phorophyte bark) can be highly heterogeneous at spatial scales and thus have significant impacts on fungal composition and the distribution of orchids [29,30]. However, the possible fluctuation of fungal–orchid associations on phorophyte trees or rocks has rarely been addressed at temporal scales. In particular, it remains unclear if the fungal richness and community composition of epiphytic orchids are temporally stable or not from a whole root lifecycle perspective.

*Pleione bulbocodioides* is a world-famous ornamental plant and an important Chinese medicinal herb (known as Shancigu) [31,32]. This species, harboring lithophytic plants with perennial habit but annually renewed leaves, roots, and pseudobulbs, allows us to monitor OMF changes with increasing root age over a 1-year period. From early summer to late autumn, we collected root samples from one lithophytic population of *P. bulbocodioides* on seven occasions and carried out high-throughput sequencings on fungal amplicons. We aimed to answer the following research questions: (1) does the OMF richness and compositions change between different root age groups of this orchid and (2) which scenario of endophytic fungal turnovers can be found and are there possible relations with the OMF?

## 2. Materials and Methods

### 2.1. Sampling and Sequencings

**Figure 1:**
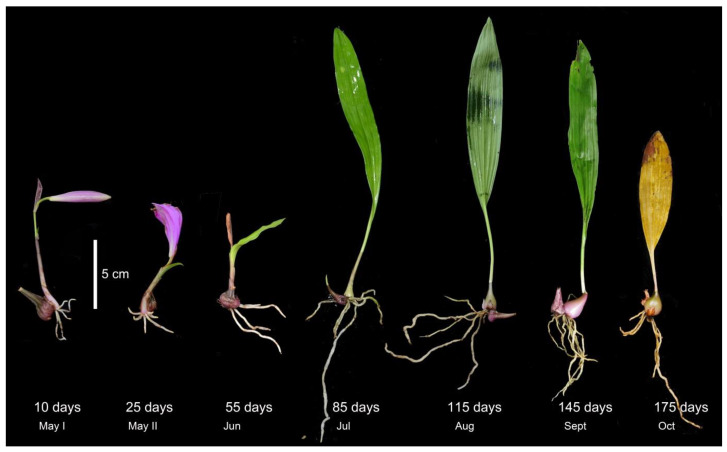
Plant morphology of *Pleione bulbocodioides* at different root ages.

*Pleione bulbocodioides* is a perennial herb that grows on mossy rocks or humus-covered soil. It is characterized by one solitary pink to pale purple flower and only one papery leaf on an annual ovoid pseudobulb. The fresh roots and one new shoot emerge at the side of the pseudobulb in late spring and then the leaf and new pseudobulb develop while the old pseudobulb shrinks gradually after flowering. The leaf and roots wither and the old pseudobulb shrivels in late October (about 175 days later after emergence), while the new pseudobulb goes into dormancy in the seasonal cold and dry conditions (Figure 1).

From early May to October 2020, we took samples 7 times from one large lithophytic population (>200 individuals) of *P. bulbocodioides* at a suburban region of Kunming, Yunnan, China. The plants were tiny-sized and the root numbers were limited (Figure 1), thus non-destructive re-samplings of the same individuals were not feasible. Six different individual plants were randomly chosen at each sampling and were treated as one root age group (3–4 roots per individual were merged into one sample). In total, 42 samples within 7 root age groups (10, 25, 55, 85, 115, 145, and 175 days) were used for molecular analyses.

**Figure 2:**
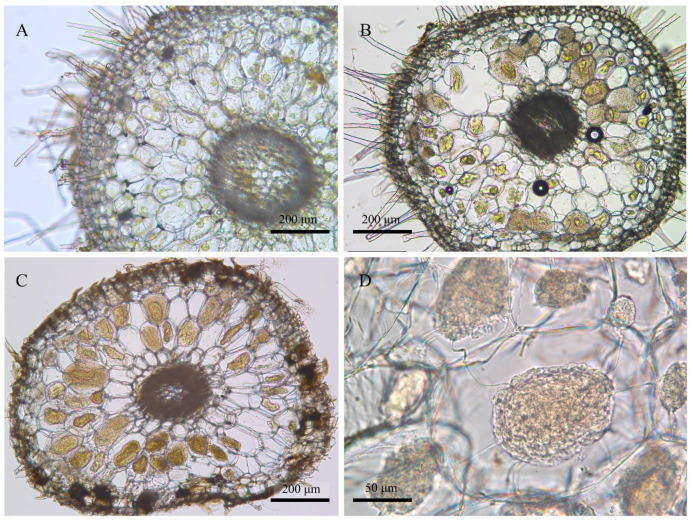
Morphology of root transections of *Pleione bulbocodioides*. (**A**) Emergent root (observed in late May, ca. 7 mm long); (**B**) elongated root (late August); (**C**,**D**) aging root (late October).

**Figure 3:**
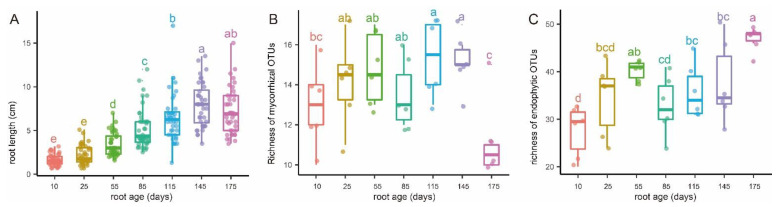
Variations of root length (**A**), putative mycorrhizal fungal richness (**B**), and endophytic fungal richness (**C**) with root age of *Pleione bulbocodioides*. Different letters (a–e) denote significant differences among the seven root age groups (post hoc Tukey–Kramer honest significant difference test, *p* < 0.05).

The roots were cleaned with tap water, transected, and checked under a light microscope to confirm the existence of pelotons (Figure 2). Forty roots were measured per sampling time to show the variations of root length at different root ages (Figure 3). The root fragments were surface-sterilized (30 s submergence in 75% ethanol, 3 min immersion in 3% sodium hypochlorite, and three rinse steps in ddH_2_O), frozen in liquid nitrogen, and then used for DNA extraction. The root epidermis was not removed since it consisted of a very thin cell layer (Figure 2). Amplicon library of the fungal ITS2-rDNA sequences was constructed using the primer pair ITS86F/ITS4 [33,34]. The primer pair was selected according to previous studies showing the usefulness for recovering a wide range of OMF [35,36,37]. Polymerase Chain Reaction (PCR) amplification, library construction, and sequencing (Illumina Mi-Seq PE-300) were conducted by Allwegene Tec. Co., Ltd. (Beijing, China).

### 2.2. Data Analyses

Sequences were assigned to each sample. Barcodes, primers, and positions with low quality were trimmed using CUTADAPT 1.0 based on a minimum Phred score of 30 (base call accuracy of 99.9%) averaged over a 50-bp sliding window [38]. Short reads (less than 170 bp), chimeras, and singletons were then excluded in Usearch 11 [39]. The reads which passed all quality control procedures were clustered into operational taxonomic units (OTUs) at a 97% identity threshold using the UPARSE-OTU algorithm [40]. The representative sequence of each OTU was blasted against the UNITE database (4 February 2020) for taxonomic assignment [41].

Operational taxonomic unit (OTU) abundance tables were generated from all of the merged sequences and OTUs and were then filtered by excluding OTUs with a low sequence abundance (when the read number ≤ 10) in subsequent analyses. The filtered OTU table was homogenized using rarefaction.py in Qiime 2 [42] according to the lowest sequence abundance in all samples (27,574 reads, OTU accumulation curves see Figure S1). Three rhizoctonia fungal lineages (Sebacinales, Tulasnellaceae, and Ceratobasidiaceae of Cantharellales) were defined as putative OMF (hereafter, OMF) of *P. bulbocodioides*, according to previous studies [43,44]. To further avoid bias caused by contamination, OTUs sporadically detected (occurred in ≤5 individual plants) were treated as opportunists and not included in fungal community analyses. Thirty-four rhizoctonia OTUs were detected and 27 occurring in more than 5 individual plants were eventually treated as OMF (GenBank Accession numbers OK165459 through OK165485, Table S1). Similarly, we detected 265 non-rhizoctonia fungal OTUs and designated 98 of them as fungal endophytes (occurring in ≥5 individual plants). To understand the phylogenetic position of the OMF OTUs, phylogenetic analysis was conducted using Maximum Likelihood (ML) algorithms in RAxML 7.2.6 [45]. GTRGAMMAI was selected as the best substitution model and statistical support was obtained using rapid nonparametric bootstrapping with 1000 replicates in the ML analysis.

Fungal (both mycorrhizal and endophytic) richness of each of the root samples were compared at different root ages using one-way ANOVAs and Tukey-Kramer post hoc comparisons. Mycorrhizal/endophytic fungal abundance data were extracted from the homogenized table and fungal presence–absence data were generated accordingly. Unconstrained principal coordinate analyses (PCoAs) of binary Bray–Curtis and unweighted UniFrac distances were then performed to investigate separations between fungal communities using the *pcoa* function from the R package “Ape” [46]. The two distances were based on presence–absence of fungal taxa and were thus more sensitive to rare taxa, with the unweighted UniFrac distance based on taxonomic relatedness, while the binary Bray–Curtis was not. To test if the fungal communities differed between root age groups, a permutational analysis of variance (PERMANOVA; [47]) was carried out using the *adonis* function in the R package “vegan” [48]. The values of R^2^ and *p* in the Permanova signify data variations explained by grouping and the statistical significance level of the difference among groups, respectively. Venn diagrams were drawn in the R package “venn” [49] to visualize the fungi shared by different root age groups.

## 3. Results

### 3.1. Root Phenology and the Variation of Mycorrhizal and Endophytic Fungal Richness with Root Age

According to phenological characters of the studied population of *P. bulbocodioides* (Figure 1), we collected the root samples from 10 May to 25 October with 15 or 30 days as intervals. Fungal pelotons were found in all root samples and those of tender roots were relatively small in size (Figure 2). The root length increased with the age and had maximum values of 14–15 cm (Figure 3A).

The 27 OMF OTUs were represented by 662,582 sequences. Among these, 12 serendipitoid, 6 sebacinoid, 5 ceratobasidioid, and 4 tulasnelloid OTUs were found and had 581,138 (87.7% of the total), 9707, 5525, and 66,212 sequences, respectively (Table S1). The richness of OMF was the lowest in 10 and 175 days-old roots and was higher in 25 to 85 days-old roots, with 115 and 145 days-old roots having significantly higher richness (Figure 3B). The average richness of OMF changed from 13 to 15.3 in most root age intervals, while a drop from 15 to 11 was observed in the last age interval (Figure 3B). The number of OMF OTUs in each individual plant varied between 10 and 17, with a median of 13.9 (Table S1).

The root-associated fungal endophytes consisted of 79 OTUs of Ascomycota, 17 of Basidiomycota, and 2 of Mortierellomycota. The mean richness of endophytic fungi increased continuously in different root age groups, except that a slight decline from 55 to 85 days was observed (Figure 3C). The number of endophytic OTUs associated with a single plant varied between 22 and 50, with an average of 36.5 (Table S1).

### 3.2. Community Compositions and the Phylogenetic Positions of Mycorrhizal Fungi

**Figure 4:**
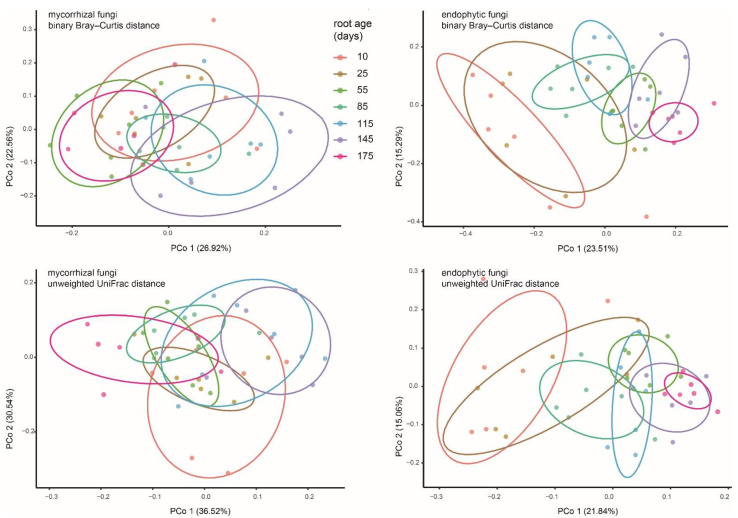
PCoA plot of binary Bray–Curtis and unweighted UniFrac distances between putative mycorrhizal and endophytic fungal communities of *Pleione bulbocodioides*. Each point corresponds to a different sample colored by compartment.

According to the results of the PCoAs (Figure 4), OMF communities were not well separated between age groups. The results of Permanova (Table 1) showed that OMF compositions between adjacent ages were mostly similar, except that significant differences were detected in the three initial stages (10, 25, and 55 days) of root growth. However, the discrepancies between the youngest roots (10 and 25 days old) and aging roots (175 days old) were not statistically supported. Endophytic fungal communities in the 10, 25, and 55 days old roots showed differences with each other, those of 85 to 145 days old roots were not significant, while the aging roots had fungal compositions significantly different from those in 145 days-old roots and the youngest roots. In both the binary Bray–Curtis and unweighted UniFrac PCoAs, the endophytic fungal compartments of the youngest roots and older roots (e.g., 145 and 175 days old) separated across the first principal coordinate, indicating that the endophytic fungi changed with root age.

**Figure 5:**
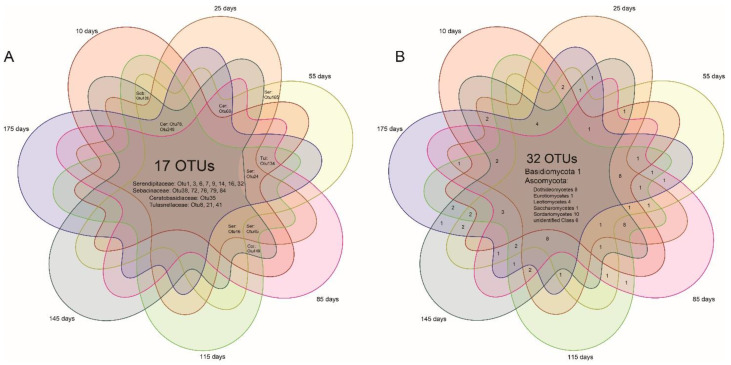
Venn diagrams for putative mycorrhizal (**A**) and endophytic (**B**) fungal OTUs of *Pleione bulbocodioides*.

Seventeen OMF OTUs were shared by 7 age groups and another 7 were shared by 5–6 age groups (Figure 5), together counting a large portion of all of the OMF (27 OTUs). Six OMF OTUs were found in more than 90% (38/42) of individual plants (serendipitoid OTU1, 3, 7, 14, tulasnelloid OTU8, and ceratobasidioid OTU35). There were 33% (32/98) endophytic OTUs shared by 7 root age groups and these mostly belonged to Ascomycota.

The MF species of *P. bulbocodioides* detected in this study were phylogenetically diverse and most of them were taxonomically undescribed (Figure S2). In particular, the four tulasnelloid OTUs clustered into the clade of *Tulasnella eremophila*, which showed large discrepancies with other species of Tulasnellaceae.

### 3.3. The Scenarios of Mycorrhizal and Endophytic Fungal Temporal Turnover of P. bulbocodioides

We assessed temporal turnover in both OMF and endophytic fungi of *P. bulbocodioides* based on OTUs detected from each age group according to the criteria of Ventre Lespiaucq et al. [3]. Five scenarios of OMF turnover were found in different time intervals, with three being partial replacement while the other two were fidelity and nested loss (Table 2). In the endophytic fungal communities, only partial replacement was found.

## 4. Discussion

### 4.1. Different Variation Trends in Mycorrhizal and Endophytic Fungal Richness

We addressed the OMF variation of one lithophytic population of *P. bulbocodioides* throughout a whole root lifecycle. Colonization by typical fungal pelotons was found at all of the sampling time points, even when the roots were only a few millimeters long (Figure 2). The OMF richness had a significant increase from root emergence to elongation (10–145 days) but was lowest in the last stage of the root lifecycle (175 days). A similar pattern has been detected in arbuscular mycorrhizal fungi of one annual herb (cheatgrass), where colonization drops dramatically with root aging after an earlier peak [50]. In contrast, the endophytic fungi had the highest richness in 175 days-old roots. This indicates that the endophytes have occupied more ecological niches in the aging roots when mycorrhizal activities decreased. One recent study also found that species of *Bletilla* have higher fungal richness from March to May, when the plants have high physiological activity [51]. In some root-persistent perennial orchids (such as *Neottia ovata*), the fungal richness is not significantly affected by time and aboveground phenological variations [25]. The coincident turnovers of fungal richness, growth, and vitality of roots implies co-adaptations of mycorrhizas and orchid hosts.

### 4.2. The Relative Constant OMF Assemblages in P. bulbocodioides

Previous studies have found that mycorrhizal colonization intensities, fungal peloton morphology, and fungal effectiveness in seed germination show seasonal changes in several terrestrial orchids [52,53,54]. Orchids with perennial belowground structures have temporal OMF-change patterns that differ by species. The fungal community of *N. ovata* and *Bletilla* spp. change significantly over time, but those of *Corallorhiza maculata*, *Paphiopedilum spicerianum,* and *Cypripedium calceolus* are not sensitive to seasonal changes [23,25,51,55]. Since fungal migrations across old and young tissues are more difficult in orchids with annual roots, they are inclined to recruit fungi from the soil species pool and thus have more frequent fungal changes [8,24,25,56,57]. For example, the younger, elongated, and older roots of *Anacamptis morio* are dominated by OMF of Tulasnellaceae, Pezizaceae, and Ceratobasidiaceae, respectively and few fungi are shared by four or five time points [8].

We discovered that serendipitoid, sebacinoid, tulasnelloid, and ceratobasidioid fungi were predominant in roots of *P. bulbocodioides*, with a large portion of OMF OTUs that were root age-independent and six of the later three families had colonized 90% of the individual plants (Figure 5, Table S1). Meanwhile, differences in the OMF composition were mostly not supported by the PCoA plots and Permanova (Figure 3, Table 1). Moreover, MF fidelity (identical OMF fungal assemblages) was detected in 10 and 115 days old roots (Table 2) and indicated a temporal conservation in OMF composition of *P. bulbocodioides.* This situation was uncommon in studies of the temporal turnover of OMF [3]. Due to the low fruit-setting ratio of natural population of *P. bulbocodioides*, no seed-bearing individual was included in our study. While the same OMF compositions between florescence and leaf expanding stages (Figure 1) implied relative constant intrinsic (physiological) demands of this species, this can be partially explained by the physiological structure (especially the pseudobulb). The succulent pseudobulb of orchids can store water, carbon, and minerals and buffer environmental stresses [58,59], thereby alleviating the fungal dependencies and minimizing potential needs for a fungal switch. In contrast, the transitions from vegetative to reproductive stages in some orchids may have heavy effects on fungal mutualisms [60,61].

Our data showed that OMF compositions of *P. bulbocodioides* were not severely affected by extrinsic or environmental factors, but the endophytic fungi showed large differences with root age (Figure 3 and Figure 4, Table 2). The variable endophytic fungal communities have reflected the existence of seasonal environmental changes (e.g., temperature, humidity or precipitation, or surrounding plants) in the habitat of this natural population. Temporal changes of abiotic factors can stimulate mycorrhizal fungal turnover in natural habitats [4,23,62], thus are worthy of further investigation in epiphytic plants.

The ubiquitous OTUs detected from plants of *P. bulbocodioides* would have fulfilled their fundamental needs and be keystone OMF of this species, while other randomly associated OTUs might provide supplementary effects, as suggested in previous studies [36,63]. Our previous work [44] has detected the serendipitoid, tulasnelloid, and ceratobasidioid fungi from *P. bulbocodioides* (based on six individuals). Here, high-throughput data from 42 individuals again confirmed these rhizoctonia lineages as keystone OMF of *P. bulbocodioides* and indicated a low degree of fungal specificity for this orchid. The co-existence of the three rhizoctonia fungal families has been reported in multi-clades of Orchidaceae [43,64,65,66,67]. These fungi may have different nutrient utilization strategies [68,69,70,71] and cause synergic effects in mycorrhizal symbiosis but needs to be further confirmed using experimental data [72]. Furthermore, the tulasnelloid fungi reported previously [44] showed divergences with the OMF detected in this study (Figure S2). One possible reason for this is the efficiencies of different sequencing strategies in fungal detection. The OMF were investigated previously [44] using Sanger sequencings of ITS1-5.8S-ITS2 region, while Next Generation Sequencing (NGS) data of the ITS2 was used in this study. Another important consideration is that individuals in the previous investigation [44] were transplanted from the wild when the pseudobulbs were dormant and had to recruit available fungi from the greenhouse with root refreshing.

## 5. Conclusions

For the first time, we found that a persistent core of OMF exists throughout one root lifecycle of a lithophytic orchid, *P. bulbocodioides*. The OMF core consisted of 17 OTUs of Sebacinales and Cantharellales and colonized the plant host rapidly (commencing at root emergences). Moreover, different temporal changes in species richness and compositions were found in the endophytic fungi of this orchid and possible reasons for these turnover scenarios were discussed. These findings broaden our knowledge of orchid mycorrhizal associations and their temporal changes and also provide practical information for conservation of the epiphytic orchids. Future research should further characterize the functional roles (synergistic, redundant, or antagonistic) of the different symbiotic fungal taxa that simultaneously associate with one orchid and also need to focus on the abiotic and possible biotic factors that may stimulate OMF turnover.

## Figures and Tables

**Table 1:** Results of the Permanova analyses based on binary Bray–Curtis distance of mycorrhizal and endophytic fungal communities at different root ages.

	Mycorrhizal Fungi		Endophytic Fungi	
	Explained Variations (R^2^)	*p* Value	Explained Variations (R^2^)	*p* Value
10 days/25 days	0.24	0.009 **	0.2	0.002 **
25 days/55 days	0.24	0.026 *	0.26	0.004 **
55 days/85 days	0.11	0.341	0.12	0.132
85 days/115 days	0.07	0.634	0.06	0.880
115 days/145 days	0.12	0.177	0.08	0.520
145 days/175 days	0.15	0.117	0.18	0.014 *
10 days/175 days	0.19	0.057	0.17	0.003 **
25 days/175 days	0.08	0.487	0.28	0.002 **

Significance codes: ** *p* < 0.01; * *p* < 0.05.

**Table 2:** The turnover scenarios of mycorrhizal and endophytic fungi in roots of *P. bulbocodioides*.

	Mycorrhizal Fungi	Endophytic Fungi
Fidelity to one OMF	no	no
Fidelity to many OMF	10 days–115 days	no
Partial replacement with constant richness	10 days–55 days; 25 days–145 days; 55 days–115 days	25 days–145 days
Partial replacement with loss	10 days–25 days, –85 days, –145 days, –175 days; 25 days–85 days, –175 days; 55 days–85 days, –145 days; 85 days–175 days; 115 days–145 days, –175 days; 145 days–175 days	25 days–55 days; 85 days–145 days; 115 days–145 days, –175 days
Partial replacement with gain	25 days–55 days, –115 days; 85 days–115 days, –145 days	10 days–25 days, –55 days, –85 days, –115 days, –145 days, –175 days; 25 days–85 days, –115 days, –175 days; 55 days–85 days, –115 days, –145 days, –175 days; 85 days–115 days, –175 days; 145 days–175 days
Nested OMF loss	55 days–175 days	no
Nested OMF gain	no	no
Narrow total replacement	no	no
Total replacement with gain, less or constant richness	no	no

## Data Availability

Publicly available datasets were analyzed in this study. This data can be found here: https://www.ncbi.nlm.nih.gov/, accessed on 17 September 2021.

## Supplementary Materials

The following are available online at https://www.mdpi.com/article/10.3390/jof7110994/s1, Figure S1: The OTU accumulation curves for each sample, plotted using the function *rarecurve* from the R package “vegan“, Figure S2: The phylogenetic tree of rhizoctonia fungi inferred from ITS2 sequences. Bootstrap values (≥50) are shown along nodes. Blue texture indicates mycorrhizal fungi of *Pleione bulbocodioides* detected in this study. Boldface and ### show the MF detected from ≥38 (90% of the total) individuals and those shared by 7 age groups. Green shows the OMF of *P. bulbocodioides* reported in one previous study [44]. Pink shows rhizoctonia fungi detected in ≤5 individuals (as opportunists) in this study, Table S1: Detailed information of each fungal OTU detected in this study.
