# Supplementary material for: Mycorrhizal Fungal Partners Remain Constant during a Root Lifecycle of Pleione bulbocodioides (Orchidaceae)
Source: J Fungi (Basel). 2021 Nov 22;7(11):994. doi: 10.3390/jof7110994 (PMC8621020; doi:10.3390/jof7110994)
Supplement: Supplementary file 1 [file jof-07-00994-s001.zip › jof-1443389-supplementary figures 1-2.pdf]

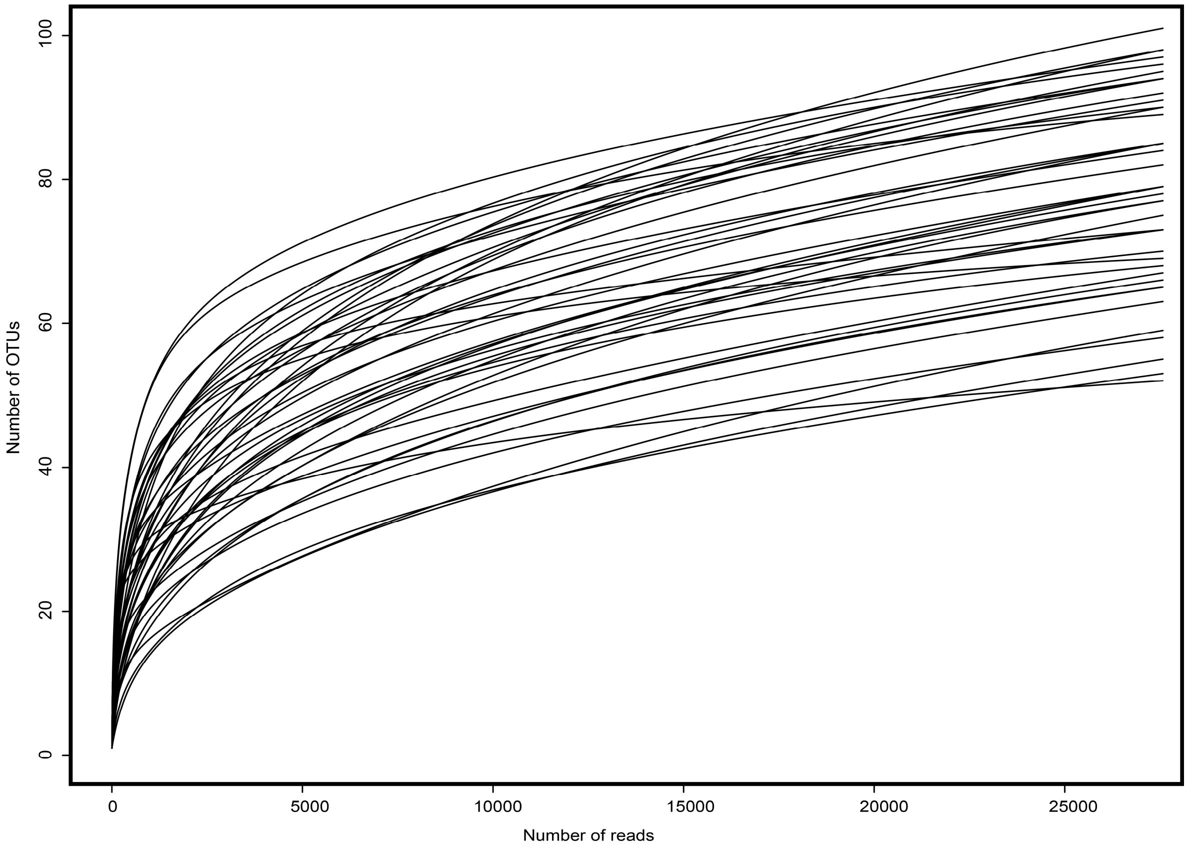

Figure S1. The OTU accumulation curves for each sample, plotted using the function *rarecurve* from the R package “vegan”.

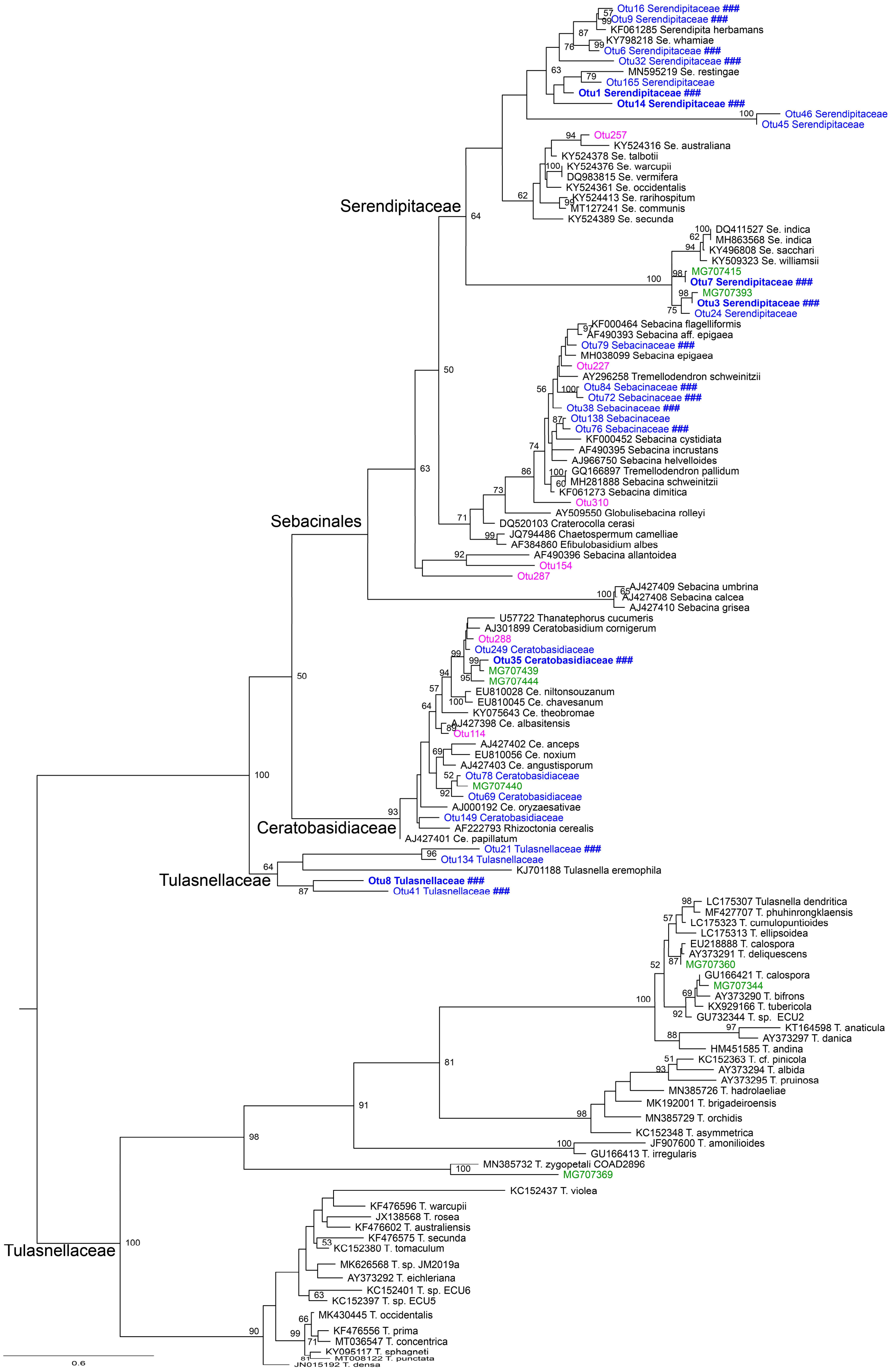

Figure S2. The phylogenetic tree of rhizoctonia fungi inferred from ITS2 sequences. Bootstrap values (≥ 50) are shown along nodes. Blue texture indicates mycorrhizal fungi of *Pleione bulbocodioides* detected in this study. Boldface and ### show the MF detected from ≥ 38 (90% of the total) individuals and those shared by 7 age groups. Green shows the OMF of *P. bulbocodioides* reported in one previous study [44]. Pink shows rhizoctonia fungi detected in ≤ 5 individuals (as opportunists) in this study.
